# *NDST4* Is a Novel Candidate Tumor Suppressor Gene at Chromosome 4q26 and Its Genetic Loss Predicts Adverse Prognosis in Colorectal Cancer

**DOI:** 10.1371/journal.pone.0067040

**Published:** 2013-06-25

**Authors:** Sheng-Tai Tzeng, Ming-Hong Tsai, Chi-Long Chen, Jing-Xing Lee, Tzu-Ming Jao, Sung-Liang Yu, Sou-Jhy Yen, Ya-Chien Yang

**Affiliations:** 1 Department of Clinical Laboratory Sciences and Medical Biotechnology, College of Medicine, National Taiwan University, Taipei, Taiwan; 2 Department of Surgery, Cardinal Tien Hospital, New Taipei City, Taiwan; 3 School of Medicine, Fu-Jen Catholic University, New Taipei City, Taiwan; 4 Department of Pathology, Taipei Municipal Wan Fang Hospital and Taipei Medical University, Taipei, Taiwan; 5 Department of Laboratory Medicine, National Taiwan University Hospital, Taipei, Taiwan; National Cancer Institute, National Institutes of Health, United States of America

## Abstract

**Background:**

Genomic deletion at tumor suppressor loci is a common genetic aberration in human cancers. The study aimed to explore candidate tumor suppressor genes at chromosome 4q25-q28.2 and to delineate novel prognostic biomarkers associated with colorectal cancer (CRC).

**Methods:**

Deletion mapping of chromosome 4q25-q28.2 was conducted in 114 sporadic CRC by loss of heterozygosity study with 11 microsatellite markers. A novel candidate tumor suppressor gene, namely *NDST4*, was identified at 4q26. Gene expression of *NDST4* was investigated in 52 pairs of primary CRC tissues by quantitative reverse transcription-polymerase chain reaction. Allelic loss of *NDST4* gene was further determined in 174 colorectal carcinomas by loss of heterozygosity analysis, and then was assessed for clinical relevance.

**Results:**

One minimal deletion region was delineated between D4S2297 and D4S2303 loci at 4q26, where *NDST4* was the only gene that had markedly been downregulated in CRC tumors. By laser capture microdissection, *NDST4* RNA expression was demonstrated in colonic epithelial cells, but was undetectable in tumor cells. In total, 30 (57.7%) of 52 colorectal carcinomas showed a dramatic reduction in *NDST4* gene expression compared with matched normal mucosae. The genetic loss of *NDST4* was significantly associated with advanced pathological stage (*P = *0.039) and poorer overall survival of patients (*P = *0.036).

**Conclusions:**

*NDST4* gene is a novel candidate tumor suppressor gene in human cancer, and the loss of its function might be involved in CRC progression. In addition, the loss of heterozygosity assay, which was established to determine the allelic loss of *NDST4* gene, could be a cost-effective tool for providing a useful biomarker of adverse prognosis in CRC.

## Introduction

Colorectal cancer (CRC) is one of the most common causes of cancer deaths worldwide, and most tumors arise sporadically by a combination of discrete mutations and chromosomal alterations [1]–[3]. Despite aggressive operations supplemented with various adjuvant therapies and an increased understanding of the genetic mechanisms underlying this disorder, there has been little improvement in the survival of patients with invasive CRC [4], [5]. Although histopathological features and staging at the time of presentation remain the most important prognostic indicators, many patients with similar pathological features display considerably different clinical outcomes [6]. Therefore, the application of sensitive genetic analysis might be useful for identifying high-risk patients and then for stratifying the design of adjuvant therapy. In addition, an improved understanding of the molecular mechanisms involved in colorectal tumorigenesis may provide new biomarkers for the potential targets of therapeutic intervention and prognostic indicators for surgical intervention [7].

Chromosomal instability is the most common genetic aberration in sporadic CRC [8], [9]. Substantial studies have revealed that allelic losses on multiple regions of chromosome 4 are associated with stage progression, tumor metastasis, and shorter survival in many human cancers, indicating the presence of one or more tumor suppressor gene (TSG) loci [10]–[15]. However, few TSGs on chromosome 4 involved in CRC pathogenesis have been identified. We recently performed deletion mapping of chromosome 4 by loss of heterozygosity (LOH) study, and identified the D4S402 locus at 4q27 that exhibited the highest allelic loss frequency of 32.5% in 106 sporadic CRC (our unpublished data). In the present study, we aimed to explore CRC-associated TSGs in the adjacent region of D4S402. Two approaches were conducted: (1) fine deletion mapping at chromosome 4q25-q28.2 to delineate the region harboring TSGs, and (2) analyses of alterations (gene expression and allelic deletion) of the candidate TSGs in primary CRC tumors. In addition, the genetic loss of the candidate TSG was assessed for clinical relevance.

## Materials and Methods

### Patients and Tissue Specimens

A total of 174 patients with sporadic CRC, who underwent surgery at Cardinal Tien Hospital, Taiwan, were recruited between August 1997 and December 2008 (Table 1). Follow-ups were conducted until April 2010. All 174 patients were operated for histologically verified colorectal adenocarcinoma without preoperative chemotherapy and/or radiotherapy. Both paired tumor and adjacent normal mucosa samples were collected from each patient during surgery. In addition, adenomatous polyp tissues were collected from 57 patients who underwent colonoscopic polypectomy. All tissue specimens were immediately frozen after resection and stored in liquid nitrogen until nucleic acid extraction. All patients provided written informed consent, and the study was conducted in accordance with the Declaration of Helsinki and approved by the Institutional Review Board of Cardinal Tien Hospital, Taiwan.

**Table 1:** Association of genetic loss of *NDST4* with clinicopathological characteristics of patients with colorectal cancer.

		Allelic loss of *NDST4* a		
Characteristic	n	Positive	Negative	*P* value^b^
Total patients	174	53 (30.5)	121 (69.5)	
Age at diagnosis (years)				0.964^c^
Median	71.5	71	72	
Range	37–98	43–97	37–98	
Gender				0.971
Male	85	26 (30.6)	59 (69.4)	
Female	89	27 (30.3)	62 (69.7)	
Tumor location				0.695
Proximal colon	36	10 (27.8)	26 (72.2)	
Distal colon	138	43 (31.2)	95 (68.8)	
Pathological differentiation				0.516
Well	11	4 (36.4)	7 (63.6)	
Moderate	119	33 (27.7)	86 (72.3)	
Poor	44	16 (36.4)	28 (63.6)	
T stage				**0.039**
T1 and T2	24	3 (12.5)	21 (87.5)	
T3 and T4	150	50 (33.3)	100 (66.7)	
N stage				0.344
N0	98	27 (27.6)	71 (72.4)	
N1 and N2	76	26 (34.2)	50 (65.8)	
M stage				0.075
M0	139	38 (27.3)	101 (72.7)	
M1	35	15 (42.9)	20 (57.1)	
Dukes’ stage				0.083^d^
A	21	3 (14.3)	18 (85.7)	
B	65	21 (32.3)	44 (67.7)	
C	53	14 (26.4)	39 (73.6)	
D	35	15 (42.9)	20 (57.1)	
Disease recurrence^e^				0.584
Yes	31	8 (25.8)	23 (74.2)	
No	87	27 (31.0)	60 (69.0)	

a Data are n (%), unless otherwise noted.

b Pearson Chi-square test, unless otherwise noted.

c Student’s t-test.

d Linear-by-linear association chi-square test.

e Only Dukes’ stages B and C were observed.

### LOH Analysis

**Figure 1:**
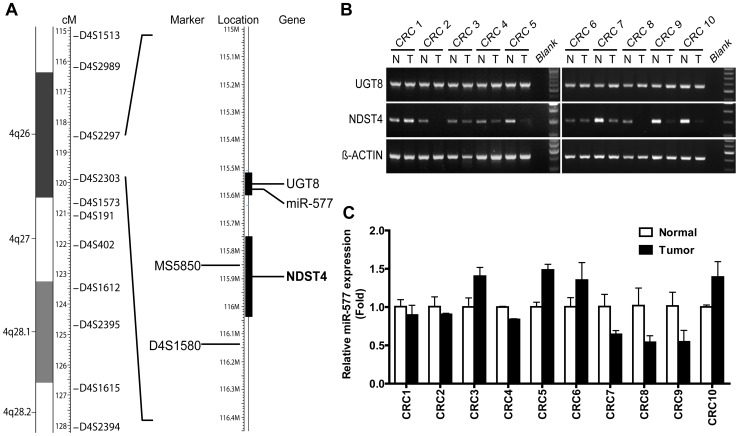
*NDST4* is identified as the candidate CRC-associated tumor suppressor gene at chromosome 4q26. **A.** Microsatellite markers used for loss of heterozygosity study. Three genes are located in the minimal deletion region delineated by D4S2297 and D4S2303. Black bars indicate *UGT8* and *NDST4* genes. *miR-577* (*MIR577*) lies in the intron of *UGT8*. **B.** Analysis of *UGT8* and *NDST4* mRNAs in tumors (T) and matched normal mucosae (N) of CRC tissues by RT-PCR. *β-ACTIN* was used as an internal RNA control. **C.** Analysis of *miR-577* expression in CRC tissues by qRT-PCR. The expression levels of tumors were normalized to those of corresponding normal mucosae. Data represent the mean ± SD.

DNA was extracted from frozen tissues by using the QIAamp DNA Mini Kit (Qiagen). For fine deletion mapping of chromosome 4q25-q28.2 (12.9 cM), LOH study with a panel of 11 microsatellites was conducted in 114 pairs of CRC tissue DNA (Figure 1A and Table 2). To further determine the allelic loss of *NDST4* gene, LOH study with two microsatellite markers, MS5850 (UniSTS:536617) and D4S1580, was conducted in 174 CRC cases (Figure 1A and Table 2). In each primer pair, the forward primer was synthesized with 6-FAM, VIC or NED fluorescent label depending on the amplicon size. PCR amplification was performed in a final volume of 6 µL by using 20 ng of DNA, 500 nM of each of respective primers, 200 µM of each dNTP, and 0.3 units of AmpliTaq Gold DNA Polymerase (Applied Biosystems). PCR was conducted under the following cycling conditions: a pre-PCR incubation step at 95°C for 15 min; followed by 35 cycles of 95°C for 15 s, 55°C for 45 s, and 72°C for 30 s; and a final extension of 72°C for 10 min. The amplified fragments were separated in 6% denaturing polyacrylamide gels on an ABI Prism 3100 Genetic Analyzer (Applied Biosystems), as described in the manufacturer’s instructions. Normal and tumor DNA pairs were compared for changes in the number of allele peaks and the peak height of each marker by using GeneScan Analysis software (Applied Biosystems). The LOH index of each normal and tumor DNA pair was calculated as previously described [16]. Briefly, the ratio of the allele peak heights calculated for each tumor sample was divided by the allele peak height ratio of the normal matching control. An LOH index of ≤0.67 or ≥1.5, representing at least a 33% decrease of a tumor allele, was indicative of allelic loss.

**Table 2:** Microsatellite markers used in loss of heterozygosity analysis.

		Amplicon	Fluorescent	Annealing
Locus^a^	Primer sequence (5′ to 3′)^b^	size (bp)	label^c^	temperature (°C)
D4S1513	F: CTGAACTGAAGTGTGTTGG	180–186	VIC	55
	R: GAAGAAAGGTGTGTCAGTAA			
D4S2989	F: TGCTGCCCAGTTGAAGG	189–217	6-FAM	55
	R: GAAAGCACTTGGCTCAGAATTG			
D4S2297	F: TAGACCCCTGGATGCAGTG	324–359	6-FAM	55
	R: ATGGAGAGAGAAAGGTTGGC			
D4S2303	F: CCACAAAGACAGAATCAATAG	198–267	6-FAM	55
	R: TCTCAACCTCCATAACTGTG			
D4S1573	F: ACATGGAGAATCTTTTAGTAGCA	98–113	NED	55
	R: CTTTTGAGATACCCCTATCAGT			
D4S191	F: AATAGGGAGCAATAAGGTGT	79–90	6-FAM	55
	R: TTTTTATTATGTTTGCTGCTC			
D4S1612	F: AAGGCTTTATTCNCTTATTGTT	160–186	NED	55
	R: GGTCCAAAGACAGGTCAAA			
D4S2395	F: TTTGATTTCCTGCAGTTGGT	216–234	NED	55
	R: TCAACACAAAACCAATGTGG			
D4S1615	F: CCTTGGGTCAGCCACATATC	115–125	VIC	55
	R: CACTCAGAACAGAAACTTGGGT			
D4S2394	F: ACTGGTATGTCCTAACCCCC	235–256	VIC	55
	R: GATCTGCAGTTGGATTCTGG			
MS5850	F: GAAACAGACCCAGCAGGATT	214–258	NED	59
	R: CCTCGTAATTGCATGAGCC			
D4S1580	F: CGTGGGGGCTATATGATTTG	267–279	VIC	59
	R: ACTCTTTAATATGTTTTGGATCTGG			

a D4S402 primer set retrieved from an ABI PRISM Linkage Mapping Set v. 2.5-MD10 (Applied Biosystems) is not shown in the table.

b “GTTCTTT” is added to the 5′ end of each reverse (R) primer to reduce “minus A” products in PCR [36].

c Fluorescent dye is labeled at the 5′ end of each forward (F) primer.

### RNA Extraction

Total RNA was extracted from the frozen tissues and 10 CRC cell lines (COLO205, HCC2998, HCT116, HCT15, HT29, KM12 and SW620 from the US National Cancer Institute; LoVo, SW48, and SW480 from the Bioresource Collection and Research Center, Taiwan) by using TRIzol reagent (Invitrogen) according to the manufacturer's instructions. The concentration and purity of RNA were determined with a Nanodrop ND-1000 spectrophotometer (Thermo Scientific), and RNA integrity was confirmed by agarose gel electrophoresis.

### Reverse Transcription-Polymerase Chain Reaction (RT-PCR)

Ten randomly selected CRC cases were used in a pilot study for gene expression. Complementary DNA (cDNA) was reverse-transcribed from total RNA (2 µg/20 µL reaction) by using the High Capacity cDNA Reverse Transcription Kit (Applied Biosystems). Reverse transcription was conducted under the following conditions: 25°C for 10 min, 37°C for 2 h, and 85°C for 5 min. The resultant cDNA was diluted 5-fold with diethylpyrocarbonate (DEPC)-treated H_2_O. Gene-specific primer sets designed spanning exons were as follows: *NDST4* forward 5′-TCTGGGAGTTACACCTCG-3′ and reverse 5′-TCTTGAGAGGCTTAGTTCTTG-3′; *UGT8* forward 5′-TTATATTATTCGTCACAATGG-3′ and reverse 5′-AAAACTAAGGTCTGACACAGT-3′; *β-ACTIN* forward 5′-ACAGAGCCTCGCCTTTGC-3′ and reverse 5′-TCATCTTCTCGCGGTTGG -3′. PCR amplification was conducted in a final volume of 25 µL by using 2.5 µL of diluted cDNA, 1 µM of each of respective primers, 250 µM of each dNTP, and 1 unit of Super-Therm Gold DNA Polymerase (Bertec Enterprise). PCR was performed under the following cycling conditions: a pre-PCR incubation step at 95°C for 10 min; 36 (*NDST4* and *UGT8*) or 28 (*β-ACTIN*) cycles of 95°C for 15 s, 55°C for 45 s, and 72°C for 45 s; followed by a final extension of 72°C for 3 min. The amplified fragments were analyzed by agarose gel electrophoresis.

### Quantitative RT-PCR (qRT-PCR)

For the quantification of *NDST4* RNA expression in 52 pairs of primary CRC tissues and 57 polyps, a TaqMan Gene Expression Assay (Hs00224024_m1, Applied Biosystems) was used with a reference gene *TBP* (TATA box-binding protein, Hs00920497_m1) as a control for RNA quality and quantity. The cDNA was synthesized as mentioned in the RT-PCR section. Quantitative PCR data were captured by an ABI PRISM 7000 Sequence Detection System and analyzed by ABI PRISM 7000 SDS Software (Applied Biosystems). The reaction mixture included 5 µL of the diluted cDNA, 1 µL of a hydrolysis probe mixture, 10 µL of TaqMan Universal Master Mix (Applied Biosystems), with the addition of DEPC-treated H_2_O to a final volume of 20 µL. The reactions were conducted in duplicate under the following cycling conditions: an incubation step at 50°C for 2 min; and an enzyme activation step at 95°C for 10 min, followed by 45 cycles of 95°C for 15 s and 60°C for 1 min. The expression levels of *NDST4* in tumors, normal mucosae and polyps were normalized to the individual reference gene, *TBP*. The tumor-to-matched-normal-mucosa ratio of *NDST4* RNA expression was calculated using the comparative Ct method, 2^−ΔΔCt^ [17]. The relative expression levels of *NDST4* in the CRC cell lines were compared with a mean expression of 52 normal mucosae, which was adjusted to 1.

### microRNA 577 Expression Analysis

For the quantification of *microRNA 577* (*miR-577*) expression in 10 pairs of randomly selected primary CRC tissues, small RNA was extracted by using the mirVana™ miRNA Isolation Kit (Ambion) according to the manufacturer's instructions. The cDNA templates were prepared from total RNA by using the TaqMan microRNA Reverse Transcription Kit (Applied Biosystems), which utilizes stem-loop reverse primers. In brief, 10 ng of total RNA was mixed with 0.8 µL of the stem-loop RT primers, 0.15 µL of 100 mM dNTP, 1.5 µL of 10× RT buffer, 0.2 µL of RNase inhibitor (20 U/µL), and 1.0 µL of MultiScribe reverse transcriptase (50 U/µL). The mixture (final volume, 15 µL) was incubated at 16°C for 30 min, 42°C for 30 min, 85°C for 5 min, and then maintained at 4°C. After RT, the cDNA products, in addition to the TaqMan primers and probes, were mixed with the other PCR reagents in the PCR Universal Master Mix Kit (Applied Biosystems), and qPCR was conducted in triplicate in the ABI PRISM 7000 Sequence Detection System. The PCR conditions included initial incubation at 50°C for 2 min and denaturing at 95°C for 10 min, followed by 40 cycles of 95°C for 15 s and 60°C for 1 min. The primers used for *miR-577* (part number 4408995) and *RNU6B* (RNA, U6 small nuclear 2, 4373381) were purchased from Applied Biosystems. As an endogenous control, *RNU6B* was amplified in parallel, and the Ct value of *miR-577* was normalized to that of *RNU6B*. The expression levels of the tumors were compared with matched normal mucosae, which were adjusted to 1.

### Laser Capture Microdissection

To confirm *NDST4* RNA expression in specific cell types of primary CRC tissues, colorectal carcinoma and epithelial cells from the matched normal mucosa were collected from two CRC cases, as well as mucosa-associated lymphoid cells were collected from the third normal tissue section. Frozen sections were fixed and stained using the HistoGene LCM Frozen Section Staining Kit (Arcturus Engineering). Cells were isolated by performing laser capture microdissection with AutoPix Automated Laser Capture Microdissection System (Arcturus Engineering). After the microdissection, the cells captured on cap were immediately incubated with an extraction buffer, and total RNA was isolated using the PicoPure RNA Isolation Kit (Arcturus Engineering), according to the manufacturer's instructions.

### Statistical Analysis

The association between allelic loss of *NDST4* gene and clinicopathological variables was analyzed by the Student’s *t*-test (for age), linear-by-linear association chi-square test (for Dukes’ stages) or Pearson chi-square test (for other categorical variables). A two-sided *P* value of <0.05 was considered statistically significant. Survival time was considered the interval between surgery and the date of the last follow-up or death of disease (overall survival, OS), or the date of the last follow-up or recurrence (disease-free survival, DFS). Survival curves, estimated with the Kaplan-Meier method, were compared by the log-rank test. All statistical analyses were performed with IBM SPSS® software, version 17.0.

## Results

### NDST4 Gene is a Candidate Tumor Suppressor Gene at Chromosome 4q26

A total of 114 pairs of primary CRC tissues were used to determine the minimal deletion region at chromosome 4q25-q28.2 by using LOH analysis. Fifty (43.9%) of the 114 tumors exhibited LOH at one or more microsatellite markers. Frequent LOH, defined as occurring in more than 30% of informative tumors, was observed in four loci, including D4S2297, D4S2303, D4S402, and D4S2394. Accordingly, one minimal deletion region with a genetic length of 1.4 Mb was then delineated between D4S2297 and D4S2303, which was involved in 80.0% (40/50) of the tumors with LOH. The results demonstrate a high frequency of chromosome 4q26 loss in colorectal carcinomas, and disclose one putative TSG locus involved in CRC development.

By searching in the National Center for Biotechnology Information (NCBI) database, only two protein-coding genes, *UGT8* and *NDST4*, as well as one microRNA, *miR-577*, were shown to be located in the defined minimal deletion region. The expression of *UGT8*, *NDST4*, and *miR-577* was screened in 10 pairs of randomly selected primary CRC tissues by RT-PCR or qRT-PCR (Figure 1B and 1C). The expression levels of *UGT8* and *miR-577* in the tested tumors were compatible with their adjacent normal mucosae. The results showed that *NDST4* gene expression was evidently decreased in six of 10 tumors. The results suggest that *NDST4* might be a novel TSG located in region 4q26, which is frequently deleted in CRC.

### NDST4 Gene is Expressed in Normal Colonic Mucosae and Polyps, but is Downregulated in Colorectal Carcinomas

**Figure 2:**
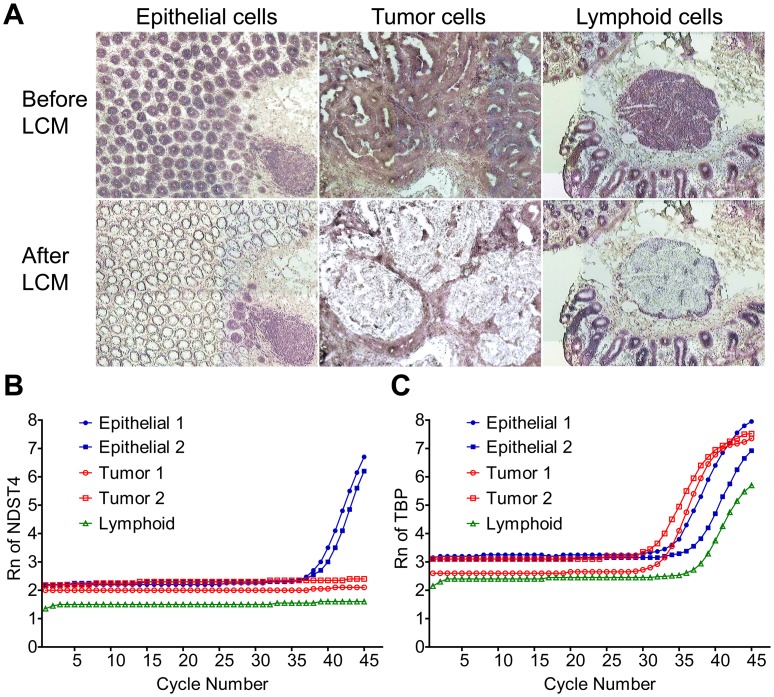
*NDST4* expression is detected in colonic epithelial cells, but not in lymphoid and tumor cells. **A.** Laser capture microdissection of different cell types from CRC tumor and matched normal mucosa sections. Representative images of HistoGene-stained slides before and after laser capture microdissection are shown. **B.** and **C.** qRT-PCR analysis of *NDST4* and *TBP* expression in the captured cells. *TBP* was used as an internal RNA control. Rn, normalized reporter signal.

**Figure 3:**
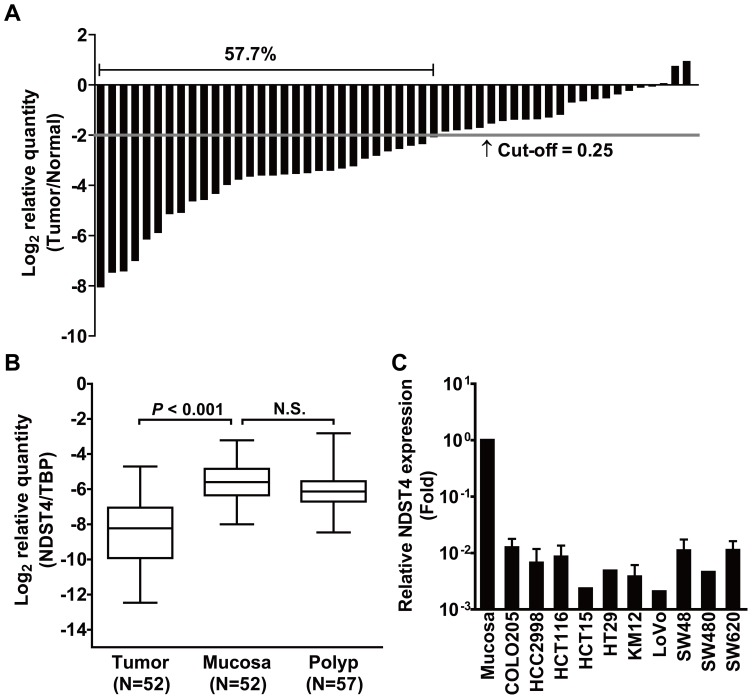
High frequency of *NDST4* downregulation is observed in primary CRC tumors and cancer cell lines. **A.** Comparison of *NDST4* expression between tumor and matched normal mucosa in 52 pairs of CRC tissues by qRT-PCR. The relative expression (Tumor/Normal) in each case is indicated by a column. **B.** Downregulation of *NDST4* expression in CRC tumors but not in adenomatous polyps. The *NDST4* expression relative to an internal control, *TBP*, is illustrated via box plot analysis. The whiskers denote the interval between the 5^th^ and 95^th^ percentiles. A significant difference between groups was tested by Student’s *t*-test (Paired, tumor *vs.* mucosa; Unpaired, mucosa *vs.* polyp). N.S., not significant. **C.** Downregulation of *NDST4* expression in all of 10 human CRC cell lines tested. The relative expression levels of CRC cells were compared with the mean expression of 52 normal colonic mucosae. Data represent the mean ± SD.

Colorectal carcinoma originates from colonic mucosa through the accumulation of genetic alterations. As a candidate of CRC-associated TSG, *NDST4* should be expressed in the colonic epithelium. Therefore, laser capture microdissection was conducted to isolate different cell types in the tissue sections, including the epithelial and lymphoid cells of normal mucosa, as well as the tumor cells of CRC, and then *NDST4* expression was determined by qRT-PCR (Figure 2). The results showed that *NDST4* mRNA was detectable in epithelial cells from both cases of normal mucosae, but neither in their paired tumor cells nor in lymphoid cells, even after 45 cycles of PCR amplification. To ascertain the downregulation of *NDST4* expression in CRC, we further analyzed 52 pairs of primary tissues. According to the Knudson two-hit hypothesis, one functional copy of a TSG may contribute to partial gene expression [18]. Therefore, a 0.25-fold decrease was defined as the threshold of significant downregulation. In total, 30 tumors (57.7%) showed an evident decrease in *NDST4* expression, compared with their matched normal mucosae (Figure 3A). In addition, *NDST4* gene expression was also determined in 57 adenomatous polyps. Unlike CRC tumors, in which *NDST4* expression was decreased substantially, the adenomas showed a similar level of expression as normal mucosae (Figure 3B). In addition, all 10 CRC cell lines studied expressed extremely low or undetectable levels of *NDST4* mRNA (Figure 3C). A dramatic reduction of *NDST4* expression in CRC sustains that *NDST4* is a novel candidate TSG, and that the loss of its function might play a role in colorectal tumorigenesis.

### Allelic Loss of NDST4 Gene is Significantly Associated with Advanced Pathological Stage and Poor Survival in CRC

To precisely determine the genetic deletion of *NDST4* in CRC, the WebSat, web software for microsatellite marker development, was used to identify short tandem repeats within *NDST4* gene [19]. A new marker, MS5850, which was designed in this study, and D4S1580 were used for LOH analysis in 174 pairs of primary CRC tissues (Figure 1A). The correlations between the genetic alteration and clinicopathological characteristics are listed in Table 1. In total, 53 (30.5%) of the 174 tumors were positive for allelic loss of *NDST4* gene. The genetic aberration was increased considerably in tumors with higher pathological stages (T3 and T4) (*P = *0.039). In addition, although not statistically significant, an increasing trend was observed in the progression of distant metastasis and Dukes’ stage (*P = *0.075 and 0.083, respectively). Nevertheless, the genetic loss was not associated with other clinicopathological features.

**Figure 4:**
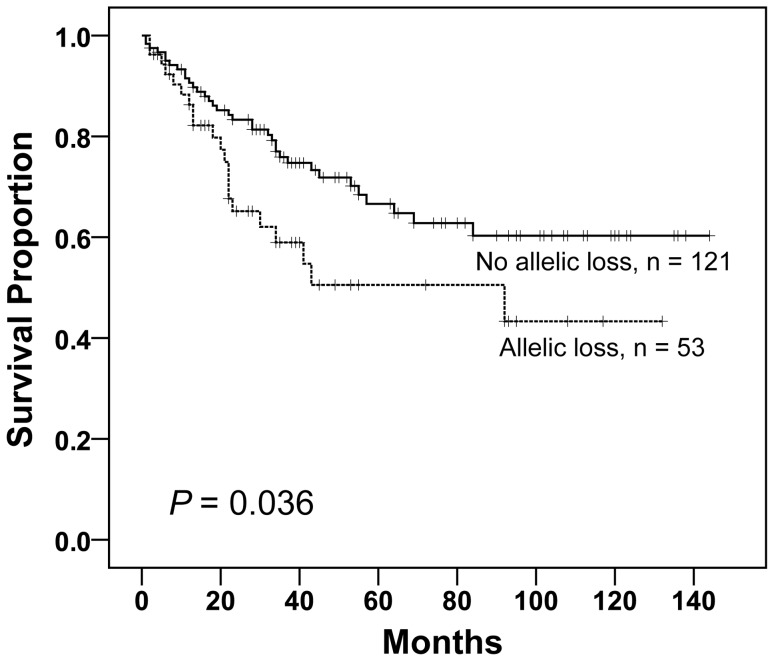
Comparison of patients’ overall survival in correlation with genetic loss of *NDST4*. Kaplan-Meier analysis was performed to compare patients with allelic loss of *NDST4* gene to the others (no allelic loss). Genetic loss of *NDST4* shows significant association with poorer survival (log-rank test).

The correlation of allelic loss of *NDST4* gene with patient survival was further evaluated. OS analysis was performed on 174 patients, of whom the OS rate was 67.8% (n = 118) with a mean survival of 94 months. By applying Kaplan-Meier survival curve analysis, the allelic loss of *NDST4* gene predicted a worse OS (*P* = 0.036) (Figure 4). Fifty-three patients with this genetic aberration had an OS of 60.4% and a mean survival of 74 months, whereas the other 121 patients had an OS of 71.1% and a mean survival of 100 months. In addition, DFS analysis was performed on 118 patients with Dukes’ stages B and C, of whom the DFS rate was 73.7% (n = 87) with a mean DFS of 104 months. Nevertheless, the genetic feature was not identified as a significant predictor of DFS for the patient group with Dukes’ stages B and C.

## Discussion

In the present study, we identified *NDST4* gene as a novel candidate TSG at chromosome 4q26, which is a common deletion region in CRC. In contrast to normal colonic mucosa with *NDST4* expression, a majority of CRC tumors and cell lines showed a dramatic reduction in gene expression. In addition, we developed an LOH assay with two microsatellite markers, and revealed that the genetic loss of *NDST4* was significantly associated with advanced pathological stage and poor survival, supporting the tumor suppressor function of NDST4 in CRC.

Despite certain molecular pathways underlying the elucidated colorectal tumorigenesis, different genetic alterations can result in a specific phenotype that is correlated with various tumor behaviors and patient outcomes [20]. Therefore, the identification of novel molecular markers is necessary to improve strategies for targeted therapies and tailored patient management. In the study, we elucidated a minimal deletion region of 1.4 Mb at chromosome 4q26 in sporadic CRC, consistent with the previous report that the frequency of allelic deletion at 4q26 was increased in colorectal carcinomas compared with adenomas [11]. Although numerous previous studies have suggested candidate TSG loci on chromosome 4 [14], [15], here we identified, for the first time, *NDST4* gene as a novel candidate TSG at 4q26. In addition, because LOH at polymorphic loci allows the expressivity of loss-of-function deletion in TSGs, this genetic study has potential diagnostic and prognostic relevance [21]. The LOH assay established in the study could be a cost-effective tool for providing a useful biomarker of adverse prognosis in CRC.

NDST4 is one member of the N-deacetylase/N-sulfotransferase (heparan glucosaminyl) (NDST) family, which is responsible for heparan sulfate (HS) biosynthesis on a core protein to form heparan sulfate proteoglycans (HSPGs) [22], [23]. HSPGs ubiquitously reside on the cell surface, inside the cell, and in the extracellular matrix [24]. The HS chains of HSPGs interact with a wide array of protein ligands such as growth factor families, and thus, contribute to the tissue structure and function during development and adult homeostasis [25], [26]. Importantly, the content and distribution of HSPGs are altered during tumorigenesis, which have been implicated in positive or negative aspects of tumor progression. For example, HSPGs function as co-receptors for growth factors and their receptor tyrosine kinases to stabilize the signaling complexes during tumor proliferation and invasion [27]. In contrast, HSPGs promote cell-cell and cell-extracellular matrix interactions and build inhibitory barriers for tumor invasion. Therefore, the decreased levels of HSPGs correlate with tumor progression [28], [29]. In the present study, the genetic loss of *NDST4* was significantly associated with advanced pathological stage, which refers to the local tumor depth of invasion in CRC, suggesting that the loss of function of *NDST4* gene might impair the modification of HS chains of specific HSPGs, leading to more invasive tumor cells through remodeling of the interaction of cell adhesion receptors and ligands.

Four different isoforms of NDSTs are identified in vertebrates. Unlike the universal gene expression of *NDST1* and *NDST2*, *NDST3* and *NDST4* transcripts are predominantly expressed during embryonic development [30], [31]. However, the expression patterns of *NDSTs* have never been illustrated in the human colon. Using RT-PCR, we found that the transcripts of four *NDSTs* were readily detectable in normal colonic mucosa, whereas only *NDST4* expression was downregulated in most of the tested CRC tumors (data not shown). According to the predicted structure of the sulfotransferase domain of NDSTs, the four different isoforms may exhibit varying substrate specificities [30]. Sheng *et al.* recently demonstrated that NDST1 performed the modification in a highly ordered manner to control the N-sulfation domains in HS, suggesting that initiated and followed N-sulfation could be conducted using different NDSTs [32]. With the relatively poor deacetylation activity of NDST4 on unmodified HS chains, NDST4 might prefer those with an initial modification by other isoforms [30]. In addition, NDSTs play a pivotal role in HS biosynthesis because NDSTs are the first participants in the sequential modification process [33]. Interestingly, because NDST4 is the only isoform that had been markedly downregulated in the tested CRC tumors (our unpublished data), the other three NDST isoforms do not seem to compensate for NDST4 deficiency in a colon-specific condition. The HS chains of HSPGs in colonic mucosa might have unique modification patterns mediated, at least in part, by NDST4 activity. Altered HSPGs resulting from the loss of function of NDST4 might vary cell and tissue arrangement, and then promote CRC pathogenesis. However, alterations in the expression of HS biosynthetic enzymes might be relatively different in a cancer-type-specific manner [34]. Fernandez-Vega *et al.* reported fairly recently that *NDST4* transcription increased in 50% of 23 infiltrating ductal adenocarcinomas studied, when it was absent in normal breast tissues [35]. Taken together, further studies are warranted to get insights into the role of NDST4 in tumor development and progression of human cancers.

In conclusion, by delineating a minimal deletion region at chromosome 4q26, this study is the first to identify *NDST4* gene as a novel candidate TSG in CRC. In addition, the genetic loss of *NDST4* might serve as a biomarker of adverse prognosis for patients with CRC. The LOH assay designed to determine the allelic loss of *NDST4* gene in the study might be used as a molecular diagnostic tool for risk stratification in individual therapeutic interventions.
